# The Application of Hollow Fiber Cartridge in Biomedicine

**DOI:** 10.3390/pharmaceutics14071485

**Published:** 2022-07-18

**Authors:** Yixuan Hou, Kun Mi, Lei Sun, Kaixiang Zhou, Lei Wang, Lan Zhang, Zhenli Liu, Lingli Huang

**Affiliations:** 1 National Reference Laboratory of Veterinary Drug Residues, Huazhong Agricultural University, Wuhan 430070, China; hyx97@webmail.hzau.edu.cn (Y.H.); mikun@webmail.hzau.edu.cn (K.M.); liuzhli009@mail.hzau.edu.cn (Z.L.); 2 MAO Key Laboratory for Detection of Veterinary Drug Residues, Huazhong Agricultural University, Wuhan 430070, China; sunlei23@webmail.hzau.edu.cn (L.S.); flyingkai@webmail.hzau.edu.cn (K.Z.); 2020302120198@webmail.hzau.edu.cn (L.W.); 3 MOA Laboratory for Risk Assessment of Quality and Safety of Livestock and Poultry Products, Huazhong Agricultural University, Wuhan 430070, China; zl-0118@webmail.hzau.edu.cn

**Keywords:** hollow fiber cartridge, in vitro model, hollow fiber infection model, hollow fiber bioreactor, hollow fiber dialyzer

## Abstract

The hollow fiber cartridge has the advantages of good semi-permeability, high surface area to volume ratio, convenient operation, and so on. Its application in chemical analysis, drug in vitro experiment, hemodialysis, and other fields has been deeply studied. This paper introduces the basic structure of hollow fiber cartridge, compares the advantages and disadvantages of a hollow fiber infection model constructed by a hollow fiber cartridge with traditional static model and animal infection model and introduces its application in drug effects, mechanism of drug resistance, and evaluation of combined drug regimen. The principle and application of hollow fiber bioreactors for cell culture and hollow fiber dialyzer for dialysis and filtration were discussed. The hollow fiber cartridge, whether used in drug experiments, artificial liver, artificial kidney, etc., has achieved controllable experimental operation and efficient and accurate experimental results, and will provide more convenience and support for drug development and clinical research in the future.

## 1. Introduction

Hollow fiber refers to a chemical fiber with a narrow cartridge-like cavity in the axial direction of the fiber. Hollow fiber materials need to be spinnable and malleable to ensure that the desired fiber bundles can be shaped and should also exhibit good water and solute permeability, high mechanical strength, sanitizability, as well as excellent biocompatibility [1]. Hollow fiber cartridge is an auxiliary experimental tool in which a certain number of hollow fiber bundles are arranged in a specially tailored lumen in a specific geometry, which can be assembled into devices systems such as ultrafiltration, dialysis, gas separation, reverse osmosis, and evaporation permeates. Hollow fiber cartridge has many advantages, such as good semi-permeability, high surface area to volume ratio, convenient operation, and so on. Its application in chemical analysis, in vitro experiment, hemodialysis, and other fields has been deeply studied. Among them, hollow fiber infection model (HFIM), bioreactor, dialyzer, and other in vitro models built with hollow fiber cartridges can more flexibly and accurately control bacteria or cell experiments, simulate the in vivo environment to a certain extent, and reduce the investment of human and material resources in the research process, which has been an area of concern for medical researchers.

Fibercell Systems, an American company, has launched a variety of different types of hollow fiber cartridges, of which polyvinylidene fluoride and polysulfone fiber cartridges are more widely used. They can be used to collect antibodies, large recombinant proteins, or PK/PD experiments in vitro. Their fiber surface area, density, and molecular weight cut-off (MWCO) are different. The non-specific binding of drugs and material should also be considered. Cellulose cartridges are also used in the research of antibacterial drugs. The use of hollow fiber cartridges has been recognized by the European Medicines Agency (EMA) and the Food and Drug Administration (FDA) as a valuable technology in the preclinical environment as a complementary tool to existing anti-tuberculosis research methods [2,3].

This review introduces the basic structure of hollow fiber cartridges and discusses the principles and applications of three important models: hollow fiber infection model for drug research, hollow fiber bioreactor for cell culture, and hollow fiber dialyzer for dialysis and filtration, which can provide some reference for the application of hollow fiber cartridge in biomedicine in the future.

## 2. Basic Structure of Hollow Fiber Cartridge

**Figure 1:**
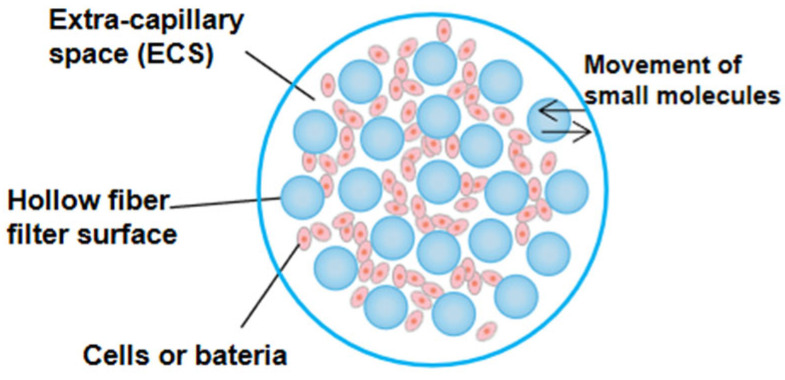
Cross section of hollow fiber cartridge, reprinted with permission from Ref. [4]. Copyright 2017, John Wiley and Sons.

Hollow fiber is an important special-shaped fiber with a hollow cross-section along the axial direction. The hollow fiber cartridge is composed of thousands of hollow fiber modules, and the filtration diameter of small cartridges in different specifications of hollow fiber cartridge is different. The chamber between the fiber and outer shell is called extra-capillary space (ECS), in which bacteria or cells are seeded (Figure 1). The medium from the central reservoir continuously recirculates inside the fibers to provide oxygen and nutritional support. Hollow fiber cartridge has the advantage of high surface area to volume ratio. Bacteria or cells can grow on the external surface of the fiber, and the liquid can be quickly and evenly distributed in the lumen.

The permeability should be considered before selecting hollow fiber cartridge. The factors that affect the permeability of hollow fiber cartridges are as follows: (1) pore diameter; (2) fiber type; (3) fiber inner diameter; (4) membrane surface area; (5) effective length; (6) arrangement, etc. In general, the pore size of the hollow fiber cartridge is required to be able to intercept cells or bacteria, allow small molecules such as gas and nutrients to pass through, and provide the most suitable growth environment for bacteria or cells. A hollow fiber cartridge is made of cellulose, polysulfone, polypropylene, or polyethylene. The choice of hollow fiber cartridge materials mainly depends on the hydrophobicity of drug. For the selection of hollow fiber, it not only needs internal circulation for nutrition, but it also must have certain mechanical properties. Cellulose hollow fibers come from a wide range of sources and are cheap, but they are not resistant to chemical reagents and have the disadvantages of low permeability and poor selective separation performance. Polysulfone hollow fiber has stable chemical properties. It has good biocompatibility, but its mechanical properties are general, and its molecular retention is relatively small. Polypropylene hollow fibers are a kind of high molecular material with large amounts and a wide range, which has excellent chemical stability, thermal insulation, high physical strength, and low price [5,6,7]. Both the 5 kD MWCO and 20 kD MWCO fibers have ten times the gross filtration rate of the equivalent cellulosic fiber for rapid nutrient and waste exchange. When the drug compound may have strong hydrophobicity and needs to be dissolved in a high concentration of solvent, or may have strong non-specific binding with polysulfone fiber, polypropylene fiber can be selected.

## 3. Construction and Application of Hollow Fiber Infection Model

### 3.1. Design Principle of Hollow Fiber Infection Model

**Figure 2:**
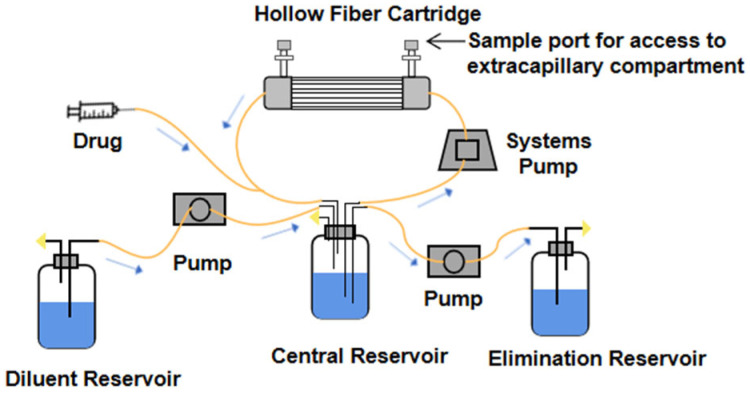
The schematic diagram of hollow fiber infection model, reprinted with permission from Ref. [4]. Copyright 2017, John Wiley and Sons.

The hollow fiber infection model (HFIM) is a closed system composed of a central reservoir, diluent reservoir, elimination reservoir, hollow fiber capillary, and pump connected by special hose (Figure 2). At present, the in vitro pharmacokinetics models of antimicrobial agents are mostly used to simulate the absorption and elimination of the kinetic process, including one-compartment models, two-compartment models, combined drug models, etc. [8]. The rate of the pump is set according to the pharmacokinetic parameters (half-life, elimination rate constant, absorption rate constant, T_max_, C_max_, etc.) of the drug. The liquid medium in the diluent reservoir containing nutrients and the drug were pumped into the central reservoir at a certain rate. In the same way, the liquid could be continuously removed from the central reservoir at a constant rate to improve the absorption. The elimination and absorption process of the drug can be accurately simulated [9].

The central reservoir is connected with the hollow fiber cartridge. The medium containing drugs enters into the fiber through peristaltic pump. The medium circulates in the central reservoir. Nutrients diffuse into ESC through the hollow fiber. Drugs and cells or bacteria in the hollow fiber cartridge are actually two dynamic processes, in order to achieve the combination of pharmacokinetics and pharmacodynamics [10]. There are sampling ports blocked by the spacer on the hollow fiber cartridge, and the sterile syringe can be used to take the samples in the hollow fiber tube. The whole set of model equipment is put into a fixed incubator to simulate the environment suitable for the growth of bacteria or cells.

HFIM is widely used in antibiotic testing [11] and antiviral testing [12]. In the 1980s, Stephen et al. used a hollow fiber sleeve containing hollow fiber for bacterial detection and constructed a model of using a permeable membrane or hollow fiber to separate two compartments for a bacterial test in vitro [11]. In the 1990s, Billello et al. used hollow fiber as an effective tool for rapid development of new drugs with anti-tuberculosis activity [13]. Blaser pointed out that HFIM can be used to simulate the pharmacokinetics models of two drugs with different half-lives [14]. The clearance rate of the in vitro system must be set according to the drugs with short half-lives and high clearance rate. By constantly replenishing the drugs with long half-lives, the problem of elimination too fast due to the short half-life of another drug can be made up. A compartment model is added to the system to simulate the in vitro experiment of different half-life drugs [15]. Based on the apparent volume of distribution (V), when the drug distribution in the body reaches dynamic equilibrium and the volume of the model, the dosage can be calculated [16]. HFIM is used to study *Mycobacterium tuberculosis*, for which the EMA issued a qualification opinion. It was developed to simulate human PK dynamics in vitro, while PD sampling of a liquid *M. tuberculosis* culture can be performed whenever required [2].

### 3.2. Characteristics of Hollow Fiber Infection Model

In recent years, many studies show that the use of HFIM to carry out research on antibacterial drugs has been widely recognized. The traditional static experiment can only monitor the change of the number of bacteria under constant drug concentration, but the constant drug concentration deviates greatly from the real drug concentration [17]. Therefore, more accurate experimental data can be obtained by using a dynamic model. Relevant studies have confirmed that pharmacokinetic/pharmacodynamic (PK/PD) parameters obtained in in vitro dynamic models are very similar to those obtained in vivo [18,19]. The in vitro models were used to simulate the pharmacokinetics of nimofloxacin. All the monitoring data fit well with the target curve, with the relative deviation below 10%. The mean relative deviation of *f*C_max_/MIC and *f*AUC_0__–24_/MIC was 5.1% and 3.3%, respectively. Meanwhile, the time distribution of concentration in vitro was reproduced by double-chamber model, which indicated that the multiphase regulation of flow rate could simulate the pharmacokinetics of nimofloxacin in vivo. It can not only simulate the process of drug concentration change in animals accurately, but also eliminate the differences among animal species [20]. Among the four dosage regimens of levornidazole, the relative deviation between PK parameters obtained from the in vitro anaerobic model and the corresponding values in vivo was generally within ±10% [21]. The t_1/2_ and C_max_ of voriconazole and caspofungin obtained by meletiadis in vitro model were similar to those in human plasma, and the AUC_0__–24_ values were similar to those in the human body (Table 1). The therapeutic effect of amphotericin B on three strains of *Aspergillus* was also consistent with that of the animal model [22,23,24,25].

Compared with the animal model, the in vitro dynamic model has several advantages: more flexible, stronger adaptability to different conditions, lower cost and so on. The significant disadvantage of animal models for PK study is the inter-individual or -species differences resulting from metabolism, which requires complex methods to transfer data from animals to people [26]. On the other hand, HFIM avoids the ethical problems of experimental animals and reduces the difficulty and risk of the experiment. For example, it is difficult to establish the in vivo infection model of *Mycoplasma gallisepticum*, and the in vitro infection model can solve this problem [27]. In addition, in vitro models allow better study of drug resistance, as the mutation frequency in in vitro studies is higher than in vivo studies. Fosfomycin resistance develops readily in vitro but less so in vivo. This discordance, at least partly, can be attributed to the function of the immune system in vivo and the lower likelihood that fosfomycin was selected in acidic environments [28]. At present, the temperature, sampling, and pump flow of the whole system can be controlled through computer to improve the experimental efficiency [9].

The components of HFIM also have certain advantages. First of all, the hollow fiber has a large surface-volume ratio, which is conducive to the adhesion and growth of bacteria or microorganisms inoculated in ECS, and ensures the growth activity of bacteria. Secondly, one of the uncontrollable problems faced by in vitro microbial experiments is contamination caused by repeated sampling, which may lead to the increase of experimental error or even experimental failure. HFIM is a closed system, drugs and organisms are stored in sealed compartment, not easy to contact with the external environment, with biological safety. Moreover, HFIM has a small volume of central reservoir. The rapid change of drug concentration in the central reservoir is achieved by adding or removing liquid medium to simulate the administration and drug elimination, which enables dynamic pharmacokinetic simulation, especially for the research with short half-life and requiring large amounts of drugs and diluents [2,10,11].

### 3.3. Application of HFIM in the Medicine

#### 3.3.1. Evaluation of Drug Effects

The study of drug effects needs to consider the dosage, times of administration, and action; complex and lasting experiments need a large number of experimental animals and economic support. In vitro studies offer the possibility to study the relationship between drug exposure and antimicrobial efficacy. With time-kill experiments, the growth and killing kinetics of a pathogen are followed over time and the observations are efficiently summarized and related to drug exposure in a PK/PD model structure with parameters estimated based on available data [29]. The HFIM can obtain pharmacokinetic and pharmacodynamic data closer to the body, so as to obtain accurate PK/PD parameters. These data can provide reference for clinical trials and reduce the risk and investment of drug research and development.

Currently, preclinical studies focused more on PK/PD studies. Static models can be used as a starting point for studies, and HFIM may be a valuable tool for evaluating PK/PD parameters. HFIM inoculated with a methicillin-susceptible strain of *Staphylococcus aureus* (ARC516) demonstrated AUC/MIC to be the index most closely associated with the activity of zoliflodacin. The PK/PD index of *f*AUC/MIC generated in the HFIM with zoliflodacin against *S. aureus* and unbound exposure magnitudes derived from in vivo neutropenic thigh models conducted in mice were subsequently utilized in the surrogate pathogen approach to help establish dose ranges for clinical development with *Neisseria gonorrhoeae* [30]. The bactericidal activity of levnidazole against *Bacteroides fragilis* by placing the HFIM in an anaerobic environment and simulating four regiments of levnidazole single-dose intravenous drip. The results showed that PK/PD parameter AUC_0__–24_ h/MIC and C_max_/MIC was greater than T MIC, indicating that the activity of the drug against *B. fragilis* was concentration-dependent [21]. In the PK/PD target study of 12 strains of clinical methicillin-resistant *S. aureus* (MRSA) strains with different characteristics, it was found that the PK/PD targets of MRSA were not affected by non-penicillin binding domain (non-PBD) substitution, and usually MIC ≥ 2 mg/L [31].

HFIM can be used in the study of pharmacodynamic mechanisms and effects because it can realize the long-time culture and the simulation of multiple conditions. The killing effect of linezolid on *M. tuberculosis* under different exposure levels was simulated in HFIM for 29 days, and it was determined that linezolid once a day had significant activity on *M. tuberculosis* in acid phase and non-replicative duration [32]. The effects of different drugs on bacteria can also be better compared in the HFIM, so as to facilitate clinical decision over the choice of appropriate antibiotics. When the effect of different drugs on *Streptococcus* in HFIM was studied, it was confirmed that linezolid, clindamycin, and penicillin had a good therapeutic effect on *Streptococcus*. In particular, continuous injection of penicillin showed a strong effect, which was generally consistent with in vivo results and provided a new method for studying the effects of *Streptococcus* inoculation [33]. There was no statistically significant difference (*p* 0.05) in maximal kill and effective concentration mediating 50% of the bacterial kill among rifampin, rifapentine, and rifabutin in the static concentration experiment. However, in the HFIM, there were significant differences in the bactericidal kill (day 0–4) of the three drugs. Due to the long culture time, drug resistance can be found in the entire *Mycobacterium avium-complex* (MAC) population. Therefore, replacing one rifamycin, due to the emergence of drug resistance, with another may not be beneficial in a clinical setting [34]. In addition, HFIM is also used to study the antibiotic exposure and bacterial killing of some novel drugs, for example gepotidacin [35] and zoliflodacin [36].

#### 3.3.2. Study of Drug Resistance

Antibiotic resistance is one of the major challenges facing global public health today. Therefore, we should re-examine the development and utilization of antibiotics and the causes of bacterial resistance and prevent the outbreak of untreatable infectious diseases. An increasing number of reports on the isolation of resistant pathogens combined with a weak antibiotic pipeline suggests that optimization of antibiotic therapy should be aimed at the suppression of resistance [37]. However, previous studies are limited by the continuous antibiotic concentration of more than 24 h, which is difficult to guide clinical rational drug use, and it is more difficult to explain the development of drug resistance from a quantitative perspective [28]. As an in vitro model, HFIM can determine when and at what dosage bacteria develop resistance and study the mechanism of resistance by related genetic testing techniques.

Dynamic models that mimic antimicrobial pharmacokinetics in vitro have been proven to be a useful tool in predicting the amplification of resistant mutants at clinically achievable antibiotic concentrations. Given that the enrichment of resistant mutants with concomitant loss in pathogen susceptibility should be concentration-dependent, concentration-resistance relationships are the methodological basis on which so-called “anti-mutant” antibiotic dosing regimens can be designed. Such a relationship was first established in an in vitro study with fluoroquinolone-exposed *S. aureus* using a dynamic model [38]. The HFIM was used to explore if the time in the mutant selection window (*T*_MSW_) is a reliable predicter of emergence of bacterial drug resistance. It is used in the study of resistance of floroquinolones [38], glycopeptides, lipopeptides [39] and oxazolidinones drugs [40].

The relationship between antibiotic exposure and the time course of bacterial resistance amplification in HFIM was studied in order to evaluate the candidate dosage regimens in treatment [41]. Dose fractionation of moxifloxacin against *M. tuberculosis* was carried out in HFIM to study the effects of dosing interval and half-life on microbial killing and drug resistance inhibition. The results showed that the decrease of the number of susceptible bacteria was related to average concentration (C_AVG_). The lowest susceptible population with 48 h interval and 4 h half-life was the largest [42]. HFIM was used to determine intravenous administration of fosfomycin to prevent the amplification of Escherichia coli subpopulations and the decrease of fosfomycin sensitivity. The results showed that for every 8 h of fosfomycin administration 1 to 2 g, the subpopulations expanded almost immediately [43]. HFIM was used to quantitatively and repeatedly analyze the resistance of ampicillin, fosfomycin, ciprofloxacin, and their combination to clinical urinary pathogenic *E. coli* CFT073. The results showed that the dual combination resulted in a significant delay in drug-resistant subsets 24–48 h after exposure. When the three-drug combination was used, the emergence of resistance was more delayed [44].

In addition, HFIM can simulate extreme dose or high inoculation density. Thus, it can better observe the emergence of drug resistance and is also conducive to the study of drug resistance mechanism. Therefore, 10^8^ cfu/mL or even higher inoculum can be given in the model to further study [45]. The emergence of drug resistance may depend on the relative density (mutation frequency) of intermediate/resistant subpopulation(s) in the total initial bacterial inoculum and the mutation tendency leading to drug resistance. When inoculated with high inoculum (10^8^ cfu/mL) of *Pseudomonas aeruginosa*, it was expected based on the mutation frequency that a resistant subpopulation existed, which was subsequently amplified following amikacin administration [46]. In the present study using dynamic in vitro HFIM, it was found that to provide effective *N. gonorrhoeae* killing and resistance suppression; zoliflodacin should ideally be administered as a sufficiently large single dose. In previous static time-kill experiments over 1–3 days, resistance to zoliflodacin was difficult to select [47]. However, using the HFIM and experiments over 7 days, zoliflodacin resistance amplification was observed from a 24-h time point [36]. The concentration time curve of fluconazole in cerebrospinal fluid was simulated by HFIM to explore and quantify the PD of sensitive, resistant subpopulations. When the dose was 800–1200 mg/d, the pattern of fluconazole resistance was “inverted U” type. The samples collected in the HFIM were sequenced. Combined with the murine model of Cryptococcus meningoencephalitis, it is shown that the replication of chromosome 1 is the potential mechanism leading to the development of fluconazole resistance of Cryptococcus [48].

#### 3.3.3. Evaluation of Combination Regimen

In clinical treatment, the single-drug regimen is facing a series of problems such as drug resistance, insufficient efficacy, and large side effects, so combined therapy has become an effective way to treat diseases. The combination of drugs needs to consider the various properties of the drug. HFIM can flexibly change the drug concentration, simulate a variety of dosing schemes, and obtain the dynamic change profile of microorganisms, which provides a relatively high-throughput and unrestricted method to evaluate the combination regimen. It can also be used to monitor the situation of resistant bacteria under different drug administration schemes, so as to reduce drug resistance and achieve the best treatment effect [49]. Some drug combination regimens studied in HFIM are shown in Table 2.

The use of *β*-lactamase inhibitors (BLI) and *β*-lactam (BL) antibiotics is a classic combination regimen. The exposure–response relationship for BL/BLI combinations in phase 2/3 clinical studies is difficult to characterize; therefore, in vitro and animal models are needed, preferably linked via a quantitative framework that integrates available data [50]. In the study of the combination of relebactam (REL) and meropenem, the effect of relebactam on bacterial susceptibility to imipenem was incorporated by characterizing the change in imipenem MIC in the presence of increasing concentrations of relebactam. In the presence of time-varying concentrations of relebactam, imipenem MIC becomes dynamic, thereby contributing to imipenem’s dynamic potency [51]. Ceftriaxone had a curative effect on *M. tuberculosis* even without avibactam, but avibactam significantly improved the curative effect. The killing rate of ceftriaxone–avibactam to *M. tuberculosis* was related to % T MIC, and the best target was 60% interval of administration [52]. Ceftazidime–avibactam is an effective agent for the treatment of *tuberculosis*. Avibactam requires frequent administration because of a short half-life. However, the half-life of ceftriaxone is longer and can be given intermittently. In the HFIM, this combined regimen with difference in half-life can be simulated [53]. However, one potential reason for the poor results of the combination of *β*-lactam/*β*-lactamase inhibitors is the inoculum effect. In the HFIM, the inhibitory effect of different *β*-lactam/*β*-lactamase combinations on high inoculum are often evaluated in HFIM. The study showed that high-density extended-spectrum *β*-lactamase (ESBL)-producing bacteria can be inhibited by the optimized dose regimen *β*-lactam/*β*-lactamase inhibitor combination [54].

Considerable pathophysiological disturbances often occur in critically ill patients and may lead to changes such as augmented renal clearance (ARC). ARC usually leads to significant changes in antibiotic pharmacokinetics [55] especially for antibiotics eliminated by the kidney, such as piperacillin and tobramycin, which leads to a significant reduction in exposure compared with the same dose of antibiotics observed in patients with normal renal function. Since the HFIM is an in vitro system, it lacks the immune system and can be used to predict the activity of antibiotic regimens in immunocompromised patients. It can also be combined with the mechanism model to predict the effect of different administration schemes [56]. In the study of the bactericidal effect of levofloxacin and ceftazidime on *P. aeruginosa*, it was also found that the inhibitory effect of the combination regimen on drug resistance in patients with normal renal function and renal insufficiency was different, and nodes of drug resistance could also be observed in the in vitro model [16].

**Table 2:** Drug Combinations regimen obtained from HFIM.

Bacteria	Combination Regimen	Effect	Reference
*M. tuberculosis*	rifampicin(100 mg/d) + moxifloxacin(100 mg/d)	reduce drug exposure for drug resistance inhibition	[57]
	ceftriaxone(100 mg/kg) + avibactam(15 mg/mL)	shortening the treatment time of children with disseminated tuberculosis	[49]
*A. baumannii*	ampicillin-sulbactam(8/4 g/8 h) + meropenem(2 g/8 h)+polymyxin B(1.43 mg/kg/12 h)	rapid (96 h) eradication of *A. baumannii*	[58]
*K. pneumoniae*	amikacin(300 mg/L) + fosfomycin(1200 mg/mL)	synergetic sterilization and resistance inhibition	[46]
*P.* *aeruginosa*	intravenous imipenem(500 mg/6 h) + REL(250 gm/6 h)	the best therapeutic effect	[52]
	meropenem(1 g/8 h,0.5 h infusion) + tobramycin(10 mg/kg/24 h)	synergetic sterilization and resistance inhibition	[59]
	piperacillin(4 g/4 h 0.5 h infusion) + tobramycin(5 mg/kg/24 h, 7 mg/kg/q24 h, 10 mg/kg/48 h, 0.5 h infusion)	synergetic sterilization and resistance inhibition	[56]
*E.* *coli*	polymyxin B(30,000 U/kg/day) + tigecycline(100 mg/12 h)	synergetic sterilization and resistance inhibition	[60]
	ceftazidime/avibactam + (2/0.5 g/8 h)aztreonam(2 g/6 h)	synergetic sterilization and resistance inhibition	[53]

## 4. Construction and Application of Hollow Fiber Bioreactor

### 4.1. Characteristics of Hollow Fiber Bioreactor

In vitro cell culture technology has become one of the indispensable tools in the field of biological products preparation and genetic engineering. Animal cell culture can be used to produce enzymes, vaccines, and monoclonal antibodies with high medical value. In the process of large-scale culture of animal cells in vitro, the most fundamental thing is to provide suitable conditions for cell growth and reduce or eliminate the impact of the external environment on cells as far as possible, so as to maintain high vitality and high expression of cells. However, when cells are cultured in vitro, their own characteristics will change. It is difficult to evaluate the behavior of normal cells or tumor cells in vitro unless the cell density and growth environment are equivalent to those in vivo.

Knazek reported a novel hollow fiber system simulating capillaries in vivo. This system can realize the exchange of nutrients. The high surface area to volume ratio of the system allows a large number of cells to grow in a simple device, so as to realize the cell culture in vitro [61]. The system of hollow fiber bioreactor for cell culture in vitro mainly consists of hollow fiber cartridge, medium container, oxygen supply, and pump. The principle and composition of the hollow fiber bioreactor is similar to the HFIM, but the arrangement of the hollow fiber bundle in the hollow fiber cartridge is different because the cell culture conditions are more stringent than the bacterial culture conditions. We should choose hollow fiber cartridges, which are more conducive to cell growth and maintain cell phenotype and function. The medium flows through the hollow fiber, and the cells are cultured in the inner or outer space of the fiber. The fiber membrane forms a semi-permeable barrier between the cells and the flowing medium, and small molecules such as lactic acid and glucose can penetrate freely. Secreted products such as exosomes, monoclonal antibodies, proteins, and other macromolecules gather in a small volume of ECS. Compared with a stirred tank bioreactor (STR) equipped with spin filter, a hollow fiber bioreactor has the advantages of small equipment volume, low economic cost, convenient operation, fast production speed, and concentration in the small-scale preparation of monoclonal antibodies. Compared with other bioreactors, the hollow fiber bioreactor has the advantages of porous materials and large surface area to volume ratio, which can significantly increase cell density and prolong culture time [62].

### 4.2. Application of Hollow Fiber Bioreactor in the Medicine

#### 4.2.1. Bioartificial Liver Bioreactor

Hepatic failure is one of the most difficult liver diseases to overcome because of its difficulty in curing, many complications, and high mortality. At present, the most effective treatment is liver transplantation. However, there are still some difficulties in liver transplantation, such as limited liver source, high risk, and high operation cost. Bioartificial liver is an effective auxiliary method for the treatment of hepatic failure. Clinical advancement of the bioartificial liver is hampered by the lack of expandable human hepatocytes and appropriate bioreactors and carriers to encourage hepatic cells to function during extracorporeal circulation [63]. Hollow fiber bioreactor is an effective artificial liver support system. The principle is blood circulation perfusion in the inner cavity of hollow fiber cartridge, and the outer cavity is used to culture hepatocytes. The hepatocytes cultured and proliferated in vitro are placed in the extracorporeal circulation device (bioreactor). When the patient’s blood (plasma) flows through the bioreactor, the nutrients in the container are exchanged to achieve the purpose of artificial liver support [64]. Ahmed created metabolically active human liver microtissue spheroids by using a hollow fiber bioreactor whose design and structural features ensure a uniform microenvironment and adequate oxygenation. Within the bioreactor, a proper oxygenation and supply of nutrients were provided to the cells. Indeed, the creation of a permissive microenvironment inside the bioreactor supported the formation and long-term maintenance of functional human liver microtissues [65]. In order to determine the most effective design of the hollow fiber bioreactor device for a bioartificial liver support system, it is found that the cross hollow fiber design can provide better solute removal and more effective metabolism in the comparison of interwoven-type bioreactor (IWBAL) and the dialyzer-type bioreactor (DBAL) [66].

The hollow fiber bioreactor has broad prospects and unique advantages in the application of in vitro liver support system. However, there are still some problems such as blocking the wall pore of hepatocytes and being unable to preserve for a long time. Therefore, more attention will be paid to the design of more efficient and feasible models for clinical treatment in the future. In order to solve the problem of clinical liver disease, we need to maximize the utilization of space efficiency and optimize the transport efficiency of nutrients.

#### 4.2.2. Other Applications

The monoclonal antibody obtained by hybridoma cell technology has the characteristics of high purity, good specificity, and low cost. However, the monoclonal antibody obtained from ascites by traditional hybridoma cells in mice has low purity and protein impurity. The concentration of monoclonal antibody obtained in in vitro flask is relatively low. The protein-free medium TurboDoma HP.1 (THP.1) was used to produce the CB.Hep-1 monoclonal antibody (mAb) in a CP-1000 hollow fiber bioreactor. It was able to generate more than 433 mg of IgG in 43 days. The maximum antibody concentration obtained was about 2.4 mg mL^−1^, and the IgG production per day was approximately 11 mg of monoclonal antibody, which constitutes a good concentration value in comparison to the results obtained in ascitic fluid, where concentration for this hybridoma was around 3 mg/mL. It is concluded that hollow fiber bioreactor is a suitable system for hybridoma culture [67]. Legazpi et al. used a hollow fiber bioreactor that consisted of a bundle of 2565 cellulose fibers with a molecular weight cut-off value of 30 kD and a length of 13 cm. The results showed that the concentration of monoclonal antibody produced by the hollow fiber bioreactor (HFBR) was high and the time was short, but the consumption of the medium for producing monoclonal antibody (MAb) per milligram was higher (Table 3) [68,69].

The hollow fiber bioreactor is also a promising tool to expand the production of stem cells to provide enough cells [70]. Vymetalova studied the expansion of human mesenchymal stromal cells (hMSCs) derived from the umbilical cord (UC) in a large-scale automated hollow fiber bioreactor to achieve the treatment of nervous system diseases and determines that the basic characteristics and quality of UC-hMSCs cultured in large-scale automated closed bioreactor conditions will not change [71]. Uslu et al. made the quantum bioreactor system suitable for generating large numbers of mature human monocyte-derived DCs (Mo-DCs), and they found that this in vitro cell culture method can overcome some obstacles in the production of dendritic cells for cancer vaccine because the hollow fiber bioreactor system can simulate the dense three-dimensional microenvironment of tumors in vivo [72]. Weeraphan et al. used this system to enrich the secretion of cholangiocarcinoma secretomes and found that two new secretory proteins were only found in the hollow fiber system, but not in the traditional monolayer culture system. Among the highly expressed proteins, the concentration of secretory soluble proteins in hollow fiber system was 5 times higher than that in monolayer culture system. Therefore, a hollow fiber system is very useful for preparing various proteins from low-abundance cell secretions [73]. Extracellular vesicles (EV) are nano-sized intercellular communication vehicles that participate in a multitude of physiological processes. However, low EV production yield and rapid clearance of administered EV by liver macrophages limit their potential use as therapeutic vehicles. Watson et al. found that bioreactor culture yielded ~40-fold more EV per mL conditioned medium, as compared to conventional cell culture, and the purity is about four times higher [74]. The hollow fiber method has been used to expand human primary cells under cGMP conditions. Hollow fiber bioreactors can maintain a large number of cells in a standard incubator, enrich the products, and use them for purification. In addition, the cells grown in the hollow fiber bioreactor are easy to adapt to the continuous growth of the protein-free medium without serum contaminants. On the other hand, the products prepared by bioreactor may have more diverse populations [75].

In conclusion, the hollow fiber bioreactor, with its unique geometric structure and biocompatibility, can simulate the tissue environment in vivo, and is not easily affected by the external environment, providing favorable conditions for cell growth, proliferation, and differentiation. With the deepening of research and optimization of structural materials, the hollow fiber bioreactor has been found to have potential practical value in bioremediation, biosensor, and tissue engineering, which also provides some ideas and convenience for follow-up biomedical research.

## 5. Application of Hollow Fiber Dialyzer

**Figure 3:**
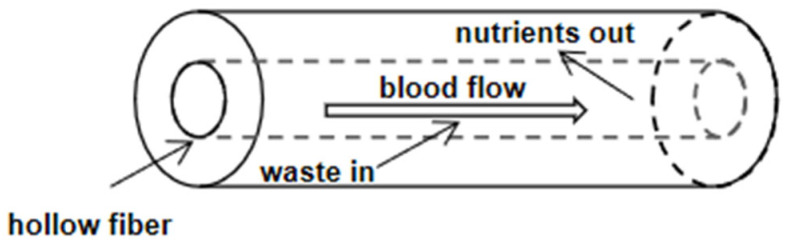
Blood capillary: the blood flows through the capillary and nutrients are delivered to the tissue while wastes are removed.

Hemodialysis is to introduce the patient’s blood into the hollow fiber dialyzer. The dialysate flows reversely with the blood on the outside of the fiber. The process of water and solute trans membrane mass transfer and separation is realized in the dialyzer. Solute clearance rate and water ultrafiltration rate are two major indexes to evaluate the performance of the dialyzer. Depending on the hierarchical structure of the circulatory system, blood vessels can distribute energy in the body, transport different nutrients or signal molecules, and remove the waste produced by cell metabolism (Figure 3). During dialysis, we should remove toxic substances in the blood of patients as much as possible and retain certain elements and plasma proteins that are beneficial to the patients. The substances lacking in patients’ blood should be supplemented from dialysate, such as serum bicarbonate, to maintain the electrolyte at an appropriate level. Meanwhile, some bacteria and their products in dialysate should be prevented from entering the blood, such as endotoxin [76].

The study found that the shape, inner diameter, and filling density of the hollow fiber bundle significantly affected the dialysate flow. The smaller diameter of the hollow fiber can lead to higher blood pressure, which makes it easier for blood to enter the hollow fiber. The shape of the hollow fiber has little effect on blood flow. Higher membrane area and pure water permeability accelerate the internal filtration, thus increasing the removal rate of high molecular weight substances [77]. The removal of urea using a urease-immobilized dialyzer was demonstrated with in vitro dialysis and showed faster removing rate of urea than a regular dialyzer by two times. The yield of urea removed by an immobilized urease dialyzer for one hour was equivalent to that by conventional dialyzer for four hours. Furthermore, the improvement in the urea clearance by the urease immobilization to a dialyzer increased with the dialysate velocity [78]. This indicated that the time needed for dialysis could be greatly reduced in the hollow fiber dialyzer immobilized with urease. L-3, 4-dihydroxyphenylalanine and human collagen type IV were coated over the outer surface of the custom-made hollow fiber with the objective of simultaneously improving biocompatibility leading to proliferation of human embryonic kidney cells-293 (HEK-293) and improving separation of uremic toxins, thereby making them suitable for bioartificial kidney application [79]. Yehl et al. have proved that the reverse-flow hollow fiber dialysis can be used for buffer liquid exchange, high impurity removal rate, low cost, and potential for longer-term continuous treatment [80].

## 6. Summary

The use and development of hollow fiber cartridge has experienced many times of innovation and optimization from the last century to now. It not only forms commercial products, but also expands and deepens its application in biomedicine. New and advanced hollow fibers with high filtration rate are adopted, which have faster material exchange and are suitable for higher density cell or bacterial culture, providing them with a more similar growth environment in vivo. Its application realizes controllable experimental operation and can obtain accurate experimental results more effectively. Moreover, the preclinical model system and its appropriate analysis have become the core part of accelerating the development of anti-infective drugs. In addition to some applications discussed in this paper, the application of hollow fiber tubes in artificial lungs, bone tissue repair, wastewater treatment, and so on is also deepening [7,81,82].

However, the hollow fiber cartridge also has some limitations. First, it cannot take account of the immune response of the host and fully reflect the human response to bacteria, cells, and drugs, which makes the experimental results conservative. Second, the hollow fiber cartridge is a disposable consumable, which is difficult to reuse and also makes the experiment more expensive. Third, cells may block the pores. Although it cannot completely replace in vivo and clinical trials, as a research supplement and development tool, it also provides new ideas for various research. In the future, the research of hollow fiber cartridges should also pay more attention to the similarity with the tissue environment in vivo, efficient reuse of hollow fiber cartridges, simplified operation, and configuration. It is believed that with the improvement of system hardware and the development of software, more potential applications of hollow fiber cartridges in biomedicine will be explored, which will bring greater hope for clinical research and disease treatment.

## Figures and Tables

**Table 1:** Pharmacokinetic parameters of amphotericin B, caspofungin, and voriconazole in humans and in vitro PK/PD model, reprinted with permission from Ref. [22]. Copyright 2012, American Society for Microbiology.

Drug (Simulated Dose [mg/kg]) and Pharmacokinetic Parameter	Avg Value in Human Plasma *^a^*	Mean Value In Vitro Model *^b^* ± SEM
Amphotericin B		
C_max_ (mg/L)	2.83	2.6 ± 0.1
t_1/2_ (h)	19.65	11 ± 1.5
AUC_0–24_ (mg·h/L)	28.98	34.52
Voriconazole		
C_max_ (mg/L)	3.62	3.7 ± 0.17
t_1/2_ (h)	6.5	5.9 ± 0.6
AUC_0–24_ (mg·h/L)	22.7	30.37
Caspofungin		
C_max_ (mg/L)	10	9.3 ± 0.25
t_1/2_ (h)	12.2	14 ± 1.25
AUC_0–24_ (mg·h/L)	97.20	120.31

*^a^* Date derived from previous clinical studies, reprinted with permission from Ref. [23]. Copyright 1996, American Society for Microbiology, [24] and reprinted with permission from Ref. [25]. Copyright 2005, American Society for Microbiology. *^b^* Obtained with the in vitro PK/PD model, reprinted with permission from Ref. [22]. Copyright 2012, American Society for Microbiology.

**Table 3:** MAb production data employing different culture techniques [68].

	i-MAb Bag *^a^* (500 mL)	T150-Flask *^a^*	HFBR *^b^*
MAb maximum concentration (mg mL^−1^)	0.8–0.74	0.030	0.220
Time to achieve the maximum MAb concentration (h)	720	50	4
Productivity (mg mL^–1^ h^–1^)	0.0001	0.0006	0.0021
Medium yield (MAb obtained/culture medium consumed) (mg mL^–1^)	0.074	0.030	0.009

*^a^* Data were reported by Legazpi, reprinted with permission from Ref. [69]. Copyright 2005, Elsevier B.V. *^b^* Data were reported by Legazpi [68].

## Data Availability

Not applicable.
